# Targeting and reducing neuronal ceramide overaccumulation in 5xFAD mice improves AD pathologies and cognitive function

**DOI:** 10.1002/alz.093451

**Published:** 2025-01-03

**Authors:** Joseph L Wilkerson, Amandine Chaix, Scott A Summers, William L Holland

**Affiliations:** ^1^ University of Utah, Salt Lake City, UT USA

## Abstract

**Background:**

Neurodegenerative disorders such as Alzheimer’s Disease (AD) are increasingly associated with irregular lipid accumulation. Dysfunction in the catabolism of sphingolipids leads to many neurodegenerative disorders but has only recently garnered interest in AD. Excess ceramide deposition has been observed in Aβ‐plaques, plasma, and cerebrospinal fluid in AD patients and AD mouse models. Ceramide‐lowering strategies have been underexplored as a treatment for AD and may prove to be a beneficial target in mitigating the disease.

**Method:**

We have used both pharmaceutical and genetic approaches to target ceramide accumulation in the 5xFAD mouse model of AD. Myriocin was used block ceramide *de novo* synthesis. The drug was administered in mice on a normal chow diet and an obesogenic pro‐ceramide high‐fat diet. As a genetic approach, we developed an inducible loss‐of‐ceramides model by overexpressing *Asah1*, the gene encoding the ceramide degrading enzyme acid ceramidase specifically in neurons. We assessed the loss of ceramides as a preventative treatment (overexpression beginning at 6‐weeks) or mature disease treatment (overexpression beginning at 30 weeks). We assessed Aβ plaques and the activation and quantity of glia by immunohistochemistry. Cognitive behavior was assessed using the Barnes maze and fear conditioning chambers. Frailty index scores were generated through clinically equivalent tests.

**Result:**

Myriocin treatment reduced ceramide accumulation in the hippocampus of 5xFAD mice. Vehicle‐treated 5xFAD mice had significant memory deficits compared to myriocin‐treated mice. The myriocin‐treated mice had smaller and fewer Aβ plaques, fewer hippocampal microglia, and a reduction in astrocyte arborization and numbers in hippocampus and cortex. Early overexpression of *Asah1* in neurons improved memory loss in fear conditioning chambers. Plaque size and number remained unchanged. However, IBA1+ microglia/monocyte and GFAP+ astrocyte activation and migration were eliminated in neuronal *Asah1* overexpressing mice. Plaques in these mice had no associated glia and glia morphology resembled wild‐type mice. Late overexpression of *Asah1* in neurons led to improved frailty index scores compared to 5xFAD mice at one year of age.

**Conclusion:**

Pharmaceutical inhibition of ceramide synthesis improved cognitive outcomes and reduced AD pathologies in 5xFAD mice. Reducing ceramides specifically in neurons in 5xFAD mice eliminated gliosis and improved cognitive function.

